# Characterizations and Assays of α-Glucosidase Inhibition Activity on Gallic Acid Cocrystals: Can the Cocrystals be Defined as a New Chemical Entity During Binding with the α-Glucosidase?

**DOI:** 10.3390/molecules25051163

**Published:** 2020-03-05

**Authors:** Na Xue, Yutao Jia, Congwei Li, Binnan He, Caiqin Yang, Jing Wang

**Affiliations:** 1 Key Laboratory of Innovative Drug Development and Evaluation, School of Pharmaceutical Sciences, Hebei Medical University, Shijiazhuang 050017, China; xuena61@126.com (N.X.); jiayutao2014@126.com (Y.J.); congwei914@163.com (C.L.); ferrysvip@126.com (B.H.); 2 Heibei Chemical and Pharmaceutical College, Shijiazhuang 050026, China

**Keywords:** gallic acid, cocrystal, α-glucosidase inhibition, docking, bioavailability

## Abstract

Cocrystallization with co-former (CCF) has proved to be a powerful approach to improve the solubility and even bioavailability of poorly water-soluble active pharmaceutical ingredients (APIs). However, it is still uncertain whether a cocrystal would exert the pharmacological activity in the form of a new chemical entity, an API-CCF supramolecule. In the present study, gallic acid (GA)-glutaric acid and GA-succinimide cocrystals were screened. The solubility, dissolution rate and oral bioavailability of the two cocrystals were evaluated. As expected, AUCs of GA-glutaric acid and GA-succinimide cocrystals were 1.86-fold and 2.60-fold higher than that of single GA, respectively. Moreover, experimental evaluations on α-glucosidase inhibition activity in vitro and theoretical simulations were used to detect whether the two cocrystals would be recognized as a new chemical entity during binding with α-glucosidase, a target protein in hypoglycemic mechanisms. The enzyme activity evaluation results showed that both GA and glutaric acid displayed α-glucosidase inhibition activity, and GA-glutaric acid cocrystals showed strengthened α-glucosidase inhibition activity at a moderate concentration, which is attributed to synergism of the two components. Molecular docking displayed that the GA-glutaric acid complex deeply entered the active cavity of the α-glucosidase in the form of a supramolecule, which made the guest-enzyme binding configuration more stable. For the GA and succinimide system, succinimide showed no enzyme inhibition activity, however, the GA-succinimide complex presented slightly higher α-glucosidase inhibition activity than that of GA. Molecular docking simulation indicated that the guest molecules entering the active cavity of the α-glucosidase were free GA and succinimide, not the GA-succinimide supramolecule.

## 1. Introduction

Improving the solubility, dissolution rate and oral bioavailability of poorly soluble active pharmaceutical ingredients (API) is one of the main challenges in the pharmaceutical industry. A pharmaceutical cocrystal is a supramolecular system constructed using an API and a biocompatible small molecule (termed cocrystal former, CCF) through non-covalent forces involving hydrogen bonds and van der Waals forces. Cocrystallization with CCF has proved to be a powerful approach to tune the stability, solubility, dissolution rate, bioavailability, and mechanical properties of the poorly water-soluble API [1,2,3,4,5].

It is generally believed that the pharmacological activity of APIs is not changed by cocrystallization with a CCF. However, how the regulatory status of cocrystals is defined during the pharmacological performance of APIs is still unsettled [6,7]. It was reported that indomethacin-based cocrystals showed different transepithelial electrical resistance (TEER) values and the ability to permeate across NCM460 cell monolayers compared to the parent physical mixture of API and CCF [8]. It seems that the introduction of CCF induces changes in the biological profiles of the API through an unclear mechanism. Therefore, the FDA maintains that the cocrystal is defined as the intermediate of the drug product [7].

Gallic acid (GA) is a phenolic compound that exists in Chinese gallnut, dogwood, pomegranate, palmate-leaf rhubarb, peony bark, tea and other plants. GA possesses various pharmacological activities, including anti-inflammatory, antioxidant, antibacterial and antiviral properties [9,10,11,12,13] and provides broad application prospects for diseases, such as cardiovascular [14] and neurological [15] diseases, diabetes [16], liver fibrosis [17] and tumors [18]. Furthermore, it was reported that GA showed an obvious inhibitory activity on α-amylase and α-glucosidase, the latter being a target protein in hypoglycemic mechanisms [19]. Consequently, GA has received much attention in the field of hypoglycemic research [20].

GA has almost no toxicity and side effects even in large doses [21]. However, it is not stable at extreme temperatures and in presence of oxygen or light [22]. Furthermore, the pharmacological activities of GA are greatly discounted owing to its large particle size and poor solubility during the absorption process [23]. To improve the solubility and dissolution rate of GA, several CCFs have been used to screen GA-based cocrystals, including succinimide, caffeine, isoniazid, etc. [24,25,26,27]. These investigations have only focused on the structural characterization and evaluations of the physicochemical properties of the cocrystals.

In the present study, we evaluated the solubility, dissolution rate and in vivo bioavailability of GA-glutaric acid cocrystals and a previously described GA-succinimide cocrystal [24]. Importantly, the effect of introduction of CCF on the biological profile was investigated through assaying the α-glucosidase inhibition activity of the two cocrystals. Powder X-ray diffraction (PXRD), differential scanning calorimetry (DSC), scanning electron microscopy (SEM), Fourier transform infrared spectroscopy (FT-IR), and nuclear magnetic resonance (NMR) were used to confirm the formation of the cocrystals. The experimental evaluations on α-glucosidase inhibition activity in vitro and molecular docking simulations were used to detect whether the two cocrystals can be recognized as a new chemical entity during binding with α-glucosidase.

## 2. Results and Discussion

### 2.1. Characterizations of the Cocrystals

#### 2.1.1. PXRD Patterns and DSC Curves

**Figure 1:**
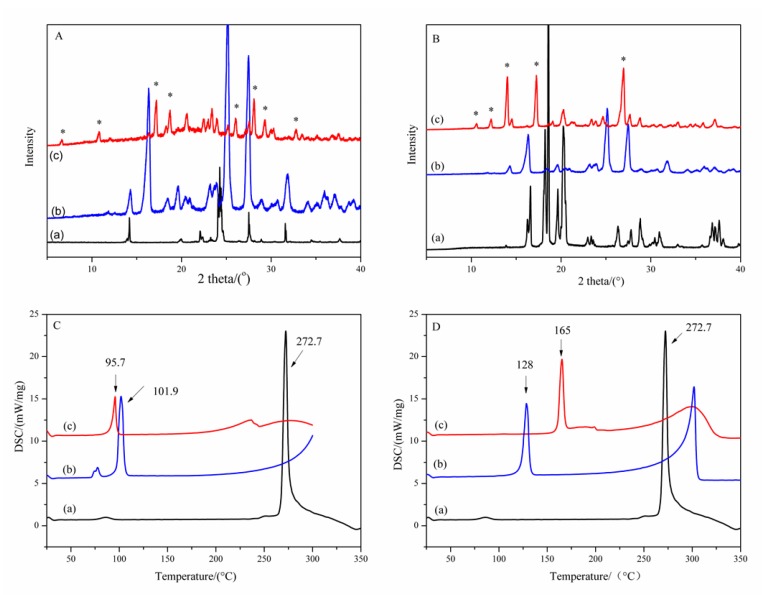
PXRD patterns (**A,B**) and DSC curves (**C,D**) of the GA-based cocrystal system (*: peaks for the new crystalloid complex) A, C: (**a**) GA; (**b**) glutaric acid; (**c**) GA-glutaric acid cocrystal in GA/glutaric acid equimolar ratio; B, D: (**a**) GA (**b**) succinimide; (**c**) GA-succinimide cocrystal in 1:2 GA/succinimide molar ratio.

The mixtures of GA and CCFs (glutaric acid and succinimide, respectively) at different GA/CCF mole ratios of 2:1, 1:1 and 1:2 were used to screen the cocrystals. PXRD patterns of samples with a GA/CCF ratio of 1:1 for the GA-glutaric acid complex and 1:2 for the GA-succinimide complex showed new peaks other than the parent GA and respective CCFs (Figure 1A,B). As seen in Figure 1A(c), new peaks appeared at 6.6°, 10.8°, 17.2°, 18.8°, 20.6°, 26.1°, 28.0°, 29.4° and 32.8° (2θ), which were apparently different from GA and glutaric acid. Meanwhile, the major peaks assigned to GA and glutaric acid disappeared, which indicated formation of a new solid phase. Suggestively, the GA-glutaric acid complex prepared by the EtOH-assisted grinding method was cocrystal in nature. Likewise, the PXRD pattern of the GA-succinimide complex exhibited obvious new characteristic peaks other than the parent GA and succinimide at 10.6°, 12.2°, 14.0°, 17.1° and 27.0°(2θ) (Figure 1B(c)), giving information on the GA-succinimide cocrystal. The DSC thermograms of GA, CCFs and their cocrystals are shown in Figure 1C,D. Melting point peaks of GA, glutaric acid and succinimide appeared at 272.7 °C, 101.9 °C and 128.0 °C, respectively; however, the new single melting peaks appeared at 95.7 °C and 165.0 °C for the GA-glutaric acid and GA-succinimide cocrystals, respectively. The emergence of a new single melting peak and the disappearance of the melting peak of the two parent components further proved the occurrence of the cocrystal.

#### 2.1.2. SEM Observation

**Figure 2:**
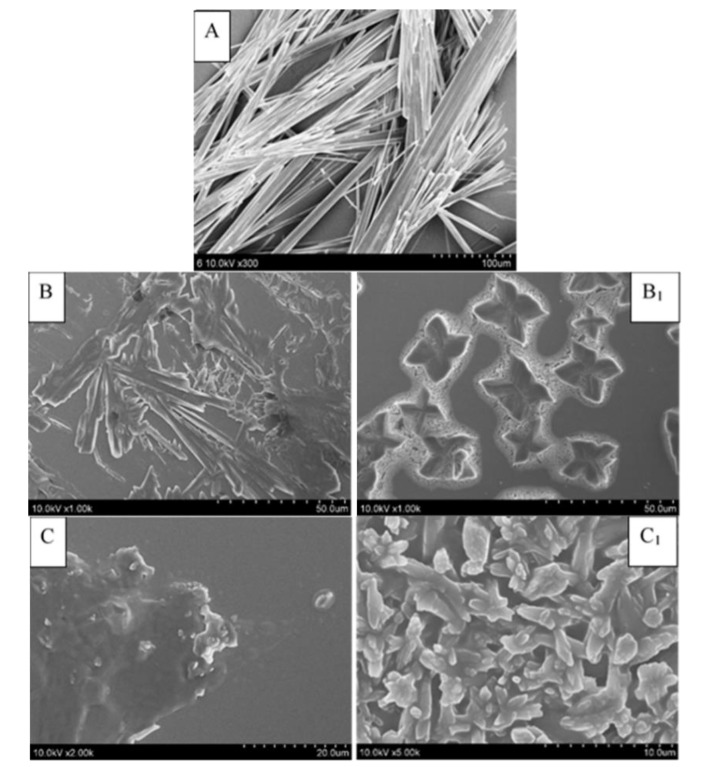
Cryo-field emission SEM photographs of GA, CCFs and the cocrystals (**A**) GA; (**B**) glutaric acid; (**B_1_**) GA-glutaric acid cocrystal; (**C**) succinimide; (**C_1_**) GA-succinimide cocrystal.

The SEM observation can be used as a supplementary technique to observe the formation of the cocrystal by comparing the morphology of API and CCF molecules with the cocrystal [28]. The SEM micrographs of GA, glutaric acid, succinimide and the two cocrystals are shown in Figure 2. Crystalline samples of pure GA showed a fibrous-shape, while the images of GA-glutaric acid and GA-succinimide cocrystals showed butterfly and flack shapes, respectively (Figure 2B_1_,C_1_). Compared with the parent components, the changed external morphology of the cocrystal provided the assisted evidence for the occurrence of the cocrystal.

#### 2.1.3. FT-IR Spectroscopy and DFT Simulation for Hydrogen Bonds in Cocrystals

**Figure 3:**
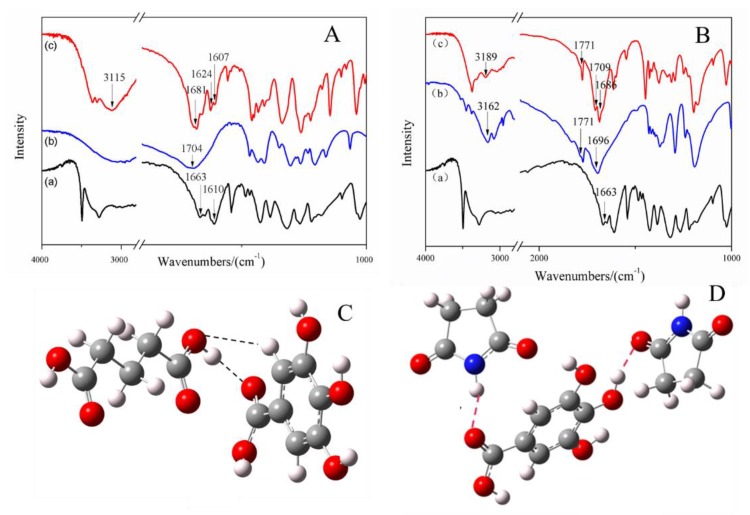
IR spectra of the GA, CCFs and their cocrystals (**A,B**) and simulated plausible models of the interactions (hydrogen bonds are marked using dashed lined) between GA and CCF cocrystals (**C,D**). Modeling was conducted using a GAUSSIAN-03 program package at the DFT B3lyp/6-31**level A, B: (**a**) GA; (**b**) CCF (glutaric acid in A and succinimide in B); (**c**) cocrystal.

Hydrogen bond formation between API and CCF in cocrystal combinations was investigated using IR spectroscopy. The FTIR spectra of GA, CCFs, and the two cocrystals are presented in Figure 3. In the spectrum of GA, we focus on the groups involving hydrogen bonding formation, including the C=O group (1663 cm^−1^) and the C–C bond in the benzene ring (1610 cm^−1^) [29]. In the spectrum of the GA-glutaric acid cocrystal, a shift of absorption peak at 1663 cm^−1^ in GA to 1681 cm^−1^ in the cocrystal was observed (Figure 3A), suggesting that the C=O group in GA was involved in the formation of a hydrogen bond with glutaric acid. In glutaric acid, no obvious peak attributed to bonded O–H was found owing to an existing general intermolecular hydrogen bond in double carboxyl organic acid. In the spectrum of the cocrystal, a broad peak at 3115 cm^−1^ appeared, indicating that the absorption of the O–H group in glutaric acid changed after cocrystal formation. In addition, the O–H group was the only hydrogen bond donor in glutaric acid. It was reasonable to confirm the occurrence of a hydrogen bond between C=O in GA and O–H in glutaric acid. Moreover, the peak at 1610 cm^−1^, assigned to a C–C bond in the benzene ring in GA, split into two peaks at 1607 cm^−1^ and 1624 cm^−1^ after cocrystal formation, which is an indicator of formation of a hydrogen bond from the group connected to the benzene ring.

In the spectrum of succinimide, peaks at 1696 cm^−1^ and 1771 cm^−1^ were attributable to the absorption of bonded and free C=O groups, respectively, and the peak at 3162 cm^−1^ was assigned to N–H absorption [30]. A shift of absorption peak at 1663 cm^−1^ in GA to 1686 cm^−1^ and a shift of 1696 cm^−1^ in succinimide to 1709 cm^−1^ in the cocrystal were observed, indicating that C=O groups in GA and succinimide were both involved in the formation of hydrogen bonds. Moreover, the absorption of the amide bond in succinimide shifted from 3162 cm^−1^ to 3189 cm^−1^ after introduction of GA (Figure 3B). It was indicated that both N–H and C=O in succinimide were involved in the formation of the weak force. The absorption at 1771 cm^−1^ of free C=O in succinimide had no change in cocrystal, indicating that free C=O was still in the cocrystal. In summary, two H∙∙∙O=C hydrogen bonds were formed between one GA molecule and two succinimide molecules in the cocrystal; one was O–H (GA)∙∙∙C=O (succinimide), and another was C=O (GA)∙∙∙H-N (succinimide).

The reasonable configurations for the two cocrystals were simulated using the DFT simulation and the GAUSSIAN-03 program [31]. The plausible models of the heterogeneous dimer in GA-glutaric acid cocrystals and the trimer in GA-succinimide cocrystals are shown in Figure 3C,D. A C=O∙∙∙H–O hydrogen bond formed between C=O in GA and O–H in glutaric acid, deduced from IR spectra, was observed in the simulated configuration of the cocrystal (Figure 3C). Furthermore, a C–H∙∙∙O=C hydrogen bond appeared between the C–H bond on the benzene ring and the C=O group in glutaric acid. The splitting of the peak of C–C on the benzene ring in the spectrum of the GA-glutaric acid cocrystal showed that the C–H bond, connected with C–C directly, was involved in the formation of the hydrogen bond. For the GA-succinimide cocrystal, the two hydrogen bonds deduced from IR spectra appeared in the simulated stable trimer configuration (Figure 3D). Molecular docking was performed using the simulated configurations of the cocrystals (*vide infra*).

### 2.2. Evaluations on Solubility, Dissolution Rate and Bioavailability

Compared with GA, the solubility of the two cocrystals was higher in all media and showed pH-independence (Figure S1A, Supplementary Material). In particular, the solubility of the GA-succinimide cocrystal increased by more than 1.5 times. A standard powder dissolution test in 0.1 mol∙L^−1^ HCl medium at 37 ± 0.5 °C was performed to compare the dissolution profiles of GA and the cocrystals (Figure S1B). The two cocrystals exhibited rapid dissolution compared with GA. The cumulative dissolution of GA reached 80% after 25 min, while the two cocrystals exceeded 85% only within 3 min. The improvement in solubility and dissolution rate induced enhanced bioavailability (Table S1). Compared with GA, the two cocrystals showed higher C _max_s, peaking up to 2.7 mg∙L^−1^ for GA-glutaric acid cocrystals and 2.2 mg∙L^−1^ for GA-succinimide cocrystals. The AUC_0–∞_ of GA-glutaric acid cocrystals and GA-succinimide cocrystals were 1.86-fold and 2.60-fold, respectively, higher than that of single GA with significant differences (*p* < 0.01).

### 2.3. α-Glucosidase Inhibitory Activity

#### 2.3.1. In Vitro Evaluation

**Figure 4:**
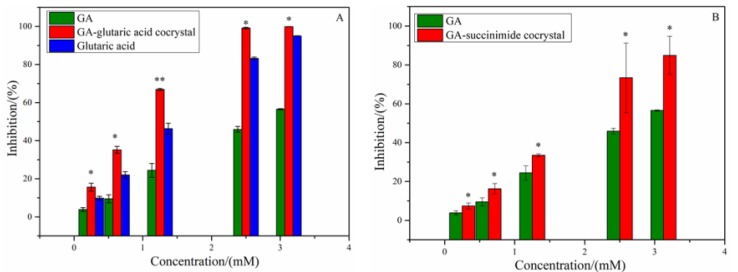
Inhibition activities of GA, CCF and cocrystals on α-glucosidase (**A**) GA-glutaric acid cocrystal system; (**B**) GA-succinimide cocrystal system. * *p* < 0.05, ** *p* < 0.01 compared with GA.

Alpha-glucosidase, known as a kind of glycoside hydrolase, locates in the brush cells of the small intestine mucosa. Oboh et al. reported that GA showed inhibitory activity on α-glucosidase [19]. To explore the change in α-glucosidase inhibitory activity of GA induced by introduction of CCF molecules, the experimental evaluation and MD simulation on α-glucosidase inhibitory activity for GA-based cocrystals were performed. The inhibitory rates of GA, glutaric acid and the two cocrystals on α-glucosidase are shown in Figure 4, and the IC_50_ values are listed in Table 1. As shown in Figure 4A, both GA and glutaric acid presented a dose-dependent α-glucosidase inhibitory activity, while succinimide showed no inhibitory activity. However, the cocrystal of GA-glutaric acid showed a higher inhibitory effect than those of the two parent components (*p* < 0.01). This indicated that cocrystallization with glutaric acid strengthened the binding of GA with α-glucosidase and produced a positive synergistic effect. Considering the GA-succinimide cocrystal system, although succinimide displayed no inhibition on α-glucosidase, the enzyme inhibition rate of the cocrystal was slightly higher than that of GA (*p* < 0.05) (Figure 4B). The occurrence of synergy between GA and CCF suggested that the introduction of CCF produced a change in binding affinity between GA and α-glucosidase by some uncertain mechanism.

#### 2.3.2. Molecular Docking

In recent years, molecular docking simulation has become an important technology in the field of computer-aided drug design. It is mainly a theoretical simulation method to study the interaction between molecules (such as ligands and receptors) and predict their binding patterns and affinity. To investigate the effect of the introduction of CCF on binding affinity between GA and α-glucosidase, molecular docking simulations were performed on the GA-CCF complex system. To clarify which form of the GA-CCF complex binds with the α-glucosidase, that is, the free GA and CCF or the GA-CCF complex, four binding patterns were designed in each system, including the binding patterns of α-glucosidase with GA, glutaric acid, free GA and CCF, and the GA-CCF supramolecule, respectively. The binding free energy of each pattern in docking, as well as experimental enzyme inhibition activity, was used to define the reasonable stable existing form of the GA-CCF complex during binding with the enzyme.

Since the docking simulations were run on an alpha-glucosidase model built on the basis of the 3D crystal structure of human glucosidase (PDB ID: 3TOP) by removing the water and acarbose molecules in the structure, the re-docking simulation allowed us to define a specific gridbox with higher fidelity. It was shown that the docked conformation (pink line) and the crystal structure conformation (yellow line) of acarbose overlapped well, and the root mean square deviation (RMSD) of the overlap was 0.75 Å, which proved the reliability and reasonability of box parameter settings in docking (Figure S2). Furthermore, many investigations confirmed that GA was shown to be a competitive inhibitor on α-glucosidase, similar to that found for acarbose, suggesting that GA and acarbose bound with α-glucosidase at similar binding sites in a manner that prevented substrate binding [32,33,34]. Based on the confirmed statements previously reported, we defined the box using the binding site of acarbose as a reference during docking. Furthermore, acarbose is a polyhydroxy compound, which is similar to GA. It was reasonable that GA and acarbose bound with α-glucosidase in the similar binding site.

**Figure 5:**
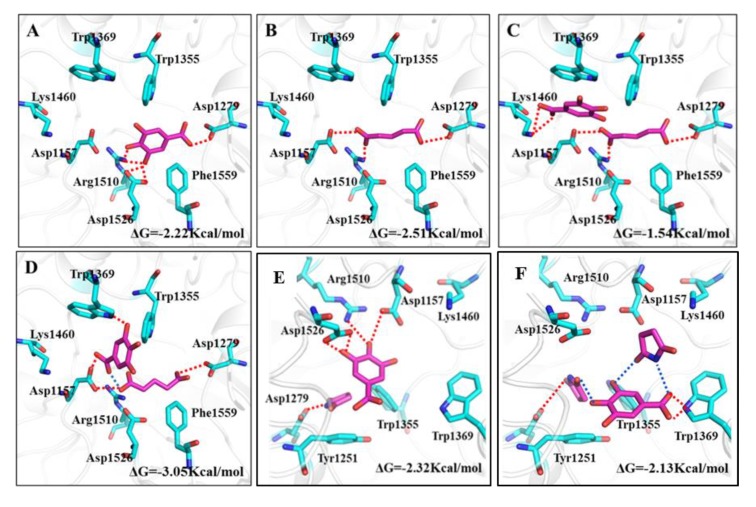
Binding patterns of GA-CCF systems with α-glucosidase. Amino acid residues and hydrogen bonding interaction in active cavity of α-glucosidase for (**A**) GA; (**B**) glutaric acid; (**C**) free GA and glutaric acid; (**D**) GA-glutaric acid complex; (**E**) free GA and succinimide; (**F**) GA-succinimide complex.

The docking results are shown in Figure 5. Owing to more hydroxyl groups in its structure, GA showed an obvious binding affinity with the polar amino acid residues in the active cavity of α-glucosidase. Attributed to a rich hydrogen bond interaction with the Asp 1279, Asp 1526 and Arg 1510 residues (red lines), GA inserted into the active cavity of α-glucosidase and achieved a stable binding conformation with a binding energy of −2.22 Kcal∙mol^−1^ (Figure 5A). Consequently, GA displayed inhibitory activity, which was consistent with the experimental inhibitory efficiency on α-glucosidase.

Glutaric acid, used as a CCF, displayed inhibitory efficiency on α-glucosidase, which was even higher than that of GA. In docking conformation of glutaric acid and α-glucosidase, carboxyl groups in glutaric acid bound with Asp 1157, Asp 1279 and Arg 1510 residues through hydrogen bonds, and the carbon chain interacted with the Phe1559 residue through hydrophobic forces (Figure 5B). The stronger binding affinity of glutaric acid with α-glucosidase than GA suggested a higher inhibitory efficiency on α-glucosidase, which was in accord with the experimental results (Figure 4A).

When a GA-glutaric acid cocrystal dissolves in solution and binds with α-glucosidase, an interesting question arises: in what form does the two components in the cocrystal enter the active cavity of the glucosidase? Figure 5C presents the binding pattern of α-glucosidase with free GA and glutaric acid. As shown, glutaric acid occupied the position of GA in the active cavity; therefore, GA had to occupy the position far from the active cavity and close to the cavity mouth. Only Lys1460 and Trp1369 residues near the cavity mouth connected with GA through a hydrogen bond and π-π interaction, which resulted in lower binding energy compared with GA. This indicated that the coexisting of glutaric acid weakened the binding of GA with amino acid residues if the free GA and glutaric acid molecules entered the cavity of α-glucosidase simultaneously. If the guest molecule entered the cavity in the form of a GA-glutaric acid supramolecule (Figure 5D), the size of the supramolecule was better matched to the size of the active cavity of α-glucosidase than single GA or glutaric acid. The abundant hydrogen bonding interactions between the GA-glutaric acid supramolecule and amino acid residues led to stable binding conformation with higher binding energy than that of GA. The GA-glutaric acid cocrystal showed a higher enzyme inhibitor activity than that of GA in the experiment. In docking, only the binding configuration between α-glucosidase and the GA-glutaric acid supramolecule showed a higher binding free energy than that of GA, indicating that the GA-glutaric acid supramolecule, not the free GA and glutaric acid, was the stable form during binding with the enzyme.

The binding energy of succinimide with α-glucosidase was too small to bind them together effectively (the binding pattern is not shown), which was consistent with the experimental result. The binding of free GA and succinimide resulted in slightly higher binding energy than that of single GA (Figure 5E). This binding conformation gave support to the experimental α-glucosidase inhibitory efficiency. However, if the guest molecule entered the active cavity of α-glucosidase in the form of a GA-succinimide supramolecule (Figure 5F), the whole supramolecule could not enter deep into the active cavity, but only bound with Trp1369 and Trp1355 residues near the cavity mouth, which resulted in slightly lower binding energy than that of GA. Considering the experimental α-glucosidase inhibitory activity and docking simulation, the guest molecule entered the active cavity of α-glucosidase in the form of free GA and succinimide, not the GA-succinimide supramolecule. Although it was difficult to find an exact relationship between the potency difference and binding energy difference in the present study, we focused on the qualitative changing trend of α-glucosidase inhibitory activity in the experiment and the binding energy in docking before and after cocrystal formation for GA, which was used to find evidence for the stable form of GA and CCF during binding with α-glucosidase.

To give support to the binding pattern between the guest molecule and α-glucosidase concluded above, ^1^H NMR characterizations in deuterated water were performed for GA, CCF and the cocrystals to detect the weak interforce between API and CCF molecules in solution media (Figure S3). Usually, the active hydrogens in the molecule do not show chemical shifts in the deuterated water solvent owing to the quick exchange of active hydrogen between the solute and the deuterated water [35]. As expected, carboxyl hydrogen and phenolic hydroxyl hydrogen were absent in the ^1^H NMR spectra of the single GA and glutaric acid (Figure S3A). However, if the hydrogen bond occurs between GA and glutaric acid, the transfer of active hydrogen between solute and solvent was inhibited; therefore, the chemical shifts assigned to active hydrogen would appear. In the ^1^H NMR spectra of the cocrystals, two new peaks at 2.89 and 2.73 ppm appeared, which were assigned to the carboxyl and phenolic hydroxyl hydrogen in GA and glutaric acid. Appearance of new chemical shifts in the ^1^H NMR spectra for the cocrystal solution suggested the occurrence of a hydrogen bond between GA and glutaric acid and provided the evidence for the binding pattern between α-glucosidase and the GA-glutaric supramolecule. However, no new peak appeared and no chemical shift was observed in the spectrum of GA-succinimide cocrystals, indicating that no hydrogen bond formed between GA and succinimide in solution (Figure S3B), which was compatible with above finding.

## 3. Materials and Methods

### 3.1. Materials

GA (purity ≥ 99%), glutaric acid (purity ≥ 98%) and succinimide (purity ≥ 98%) were purchased from J&K chemicals Ltd (Beijing, China). Alpha-glucosidase and sodium hydroxymethyl cellulose were purchased from Sigma (ALDRICH, Shanghai). p-Nitrophenol-α-d-glucoside (*p*NPG) was purchased from ACROS ORGANICS (Geel, Belgium). Acetonitrile (LC grade) was purchased from Fisher Chemical Ltd (Waltham, MA, USA). Double distilled water was used for all experiments. Male spraque-dawley rats (SD, 220 ± 10 g) were supplied by Animal Experiment Center of Hebei Medical University (SCXK 1711175, Shijiazhuang, China). The animals were performed in accordance with the Principles of Laboratory Animal Care, and approved by the Animal Care Committee of Hebei Medical University in China.

### 3.2. Methods

#### 3.2.1. Preparation of Cocrystals

Cocrystals were prepared by the EtOH-assisted grinding method. The mixtures of GA and CCFs (here glutaric acid and succinimide, respectively) at different GA/CCF mole ratios of 2:1, 1:1 and 1:2 (total mass of 1000 mg) were ground in a laboratory scale swinging ball mill (Mixer Mill GT200, Grinder tech GmbH, Beijing, China) with 35 mL of zirconia grinding jar and 1.5 cm of zirconia grinding ball at a speed of 1800 rpm. During the grinding procedure, the slurry state of the mixture was maintained through dropping 0.2 mL of EtOH every 5 min. After 30 min of grinding, the collected solid product was dried at 313 K in a vacuum oven for 5 h and stored in a vacuum desiccator.

#### 3.2.2. Characterizations

PXRD was performed on a power X-ray diffractometer (D2 Phaser, Bruker Co., Bremen, Germany) using a Cu-Kα source at 30 kV and 10 mA. The PXRD pattern was collected from 5° to 40° (*2*θ) at ambient temperature with a step size of 0.05°(*2*θ) and time per step of 0.3 s. DSC analysis was performed on a DSC 214 apparatus (NETZSCH Company, Selb, Germany). Approximately 3–5 mg of sample was accurately weighed and placed in an aluminum pan. The sample pan was heated from 20 to 400 °C at a rate of 5 °C∙min^−1^ under a nitrogen purge of 50 mL∙min^−1^. The empty aluminum pan was used as a reference. The SEM system (Hitachi S-4800, Tokyo, Japan) was used to observe the morphologies of GA, CCFs and the cocrystals. By dropping the ethanol suspension of the sample onto the electric glass slice, the precipitate on the glass slice was observed after the solvent volatilized. The FTIR spectra were recorded on a Prestige-21 infrared spectrophotometer (Shimadzu, Japan) using the KBr pellet method. The scan range was set from 400 to 4000 cm^−1^ at 2 cm^−1^ resolution. The ^1^H NMR spectra were recorded on a WIPM 400 MHz NMR spectrometer (Zhongke-Niujin, Wuhan, China) at a temperature of 293.1 K. Samples were prepared by dissolving about 5 mg of sample in 500 μL of D_2_O. The spectral width was set as 11,013 Hz. The cumulative number of times was 16, and the relaxation time D_1_ was 1 s.

### 3.3. Assays on Solubility, Dissolution Rate and Bioavailability

The apparent solubility was determined on a SPH 200B air bath shaker (Shiping Tech. Co., Ltd., Shanghai, China) at 25 °C in five different solutions (pH 1, 2, 4, 8 and water, respectively) using the shake-flask method. Excess sample was added in the solvent and the suspension was shaken for 72 h in an air bath. The suspension was filtered through a 0.45 μm Millipore filter. Then, the GA concentration was determined using HPLC (Agilent 1200, Santa Clara, CA, USA). Dissolution experiments were carried out on a RC-808D dissolution test analyzer at 37 ± 0.5 °C using 0.1 mol∙L^−1^ HCl as dissolution media at the paddle rotation speed of 50 ± 2 rpm. Approximately 50 mg of the sample (here single GA and cocrystals equivalent to 50 mg of GA) were placed in 900 mL dissolution media. Four milliliters of the suspensions were taken at appropriate intervals and immediately replaced by 4 mL of fresh dissolution media. All samples were filtered through a 0.45 μm Millipore filter. The concentration of GA in all samples were determined by HPLC. All tests were repeated thrice. Bioavailability in vivo was assayed using SD rats. The SD rats were randomly divided into three groups, termed GA, GA-glutaric acid and GA-succinimide cocrystal groups, respectively. The intragastric suspensions were prepared by suspending GA or the cocrystal (equivalent to same amount of GA) in 5% sodium carboxymethyl cellulose. Thereafter, the intragastric suspensions were orally administered to the rats at a single dose of 50 mg∙kg^−1^. An approximately 500 μL blood sample was obtained from the eye canthus and collected into 1.5 mL heparinized plastic centrifuge tubes at the set time point after intragastric administration. GA was extracted from plasma by protein precipitation. One hundred microliters of plasma was mixed with internal standard solution (20 μL) in the centrifuge tube; then, the solution of acetonitrile formic acid (2%, *v*/*v*) was added to precipitate the protein. The protein was centrifugalized for 20 min at a speed of 15,000 r∙min^−1^ at 4 °C. Then, the supernatant was taken for determining the concentration of GA by the LC-MS/MS (MS:3200 QTRAP, AB SCIEX, MA, USA, LC: Agilent1200, Santa Clara, CA, USA) method. The blood concentration-time curve was drawn by taking blood sampling time as the horizontal coordinate and GA concentration as the vertical coordinate. The pharmacokinetic parameters were calculated using DAS 2.0 software.

### 3.4. Evaluation of α-Glucosidase Inhibitory Activity

#### 3.4.1. Experimental Evaluation

The α-glucosidase inhibitory activity of GA, CCFs and the corresponding cocrystals were evaluated on a microplate spectrophotometer (Spectra Max Plus384, Molecular device, CA, USA). Briefly, accurately weighed α-glucosidase was dissolved in 0.1 mol∙L^−1^ potassium phosphate buffer (pH 6.8) to produce 50 μL of enzyme solution with a concentration of 1.0 U∙mL^−1^. Fifty microliters of enzyme solution and 50 μL of test sample were mixed in a well of a microtiter plate to incubate for 10 min at 37 °C. Three millimoles of *p*NPG in the same buffer was used as a substrate solution. After pre-incubation at 37 °C for 10 min, 50 μL of *p*NPG solution was added into above enzyme solution. The enzymatic reaction was allowed to proceed at 37 °C for 10 min, following the measurement of 4-nitrophenol absorbance at 405 nm on a microplate spectrophotometer. The solution without α-glucosidase was used as a blank. The solution without the test sample was used as a test. The solution without the test sample and α-glucosidase was used as a control. The percent inhibition of α-glucosidase was calculated using the following formula:

(1) $$\mathrm{Inhibition}\;\mathrm{rate}\;\mathrm{of}\;\alpha -\mathrm{glucosidase}\;\%\;=\;(1-\frac{{\mathrm{Abs}}_{\mathrm{sample}}{-\mathrm{Abs}}_{\mathrm{blank}}}{{\mathrm{Abs}}_{\mathrm{test}}{-\mathrm{Abs}}_{\mathrm{control}}})\text{×}100\%$$

IC_50_ values were obtained as the mean of three independent experiments, which were constructed by testing a series of concentrations (i.e., three curves were obtained, each in triplicate).

#### 3.4.2. Simulation Calculations

A density function theory (DFT) simulation was performed to obtain the supramolecular structure of the two cocrystals. The initial structures of GA and CCFs were created from the crystal structure and then optimized at the DFT (B3LYP) theoretical level combined with the 6–31G (d, p) basis set using the GAUSSIAN-03 program package. The geometries of the cocrystals were constructed and optimized at the same computational level. The DFT simulation procedure involved the first step of the configuration optimization and the second step of the frequency calculation. The nature of these supramolecular geometries as true minima was confirmed by the absence of an imaginary frequency.

A molecular docking simulation was performed to investigate the binding interaction between α-glucosidase and guest molecules. To clarify the possible binding mechanism of the cocrystal with α-glucosidase, four binding patterns were designed in each cocrystal system, including the binding of α-glucosidase with (i) GA, (ii) CCFs, (iii) free GA and CCF (no weak interaction force between GA and CCF), and (iv) the GA-CCF supramolecule (existing weak interaction force between GA and CCF). The crystal 3D structure (PDB ID: 3TOP) of human glucosidase was obtained from the PDB database (https://www.rcsb.org/). Water molecules and acarbose molecules in the structure of human glucosidase were removed and the hydrogens were added using Discovery 4.0 software. The Sybyl 2.0 software was used to optimize the structures of the small molecule GA and CCFs in the Tripos force field. The structures of the two cocrystals were constructed using DFT simulations. Auto Docking Tools 1.5.6 was used to add a Gasteiger charge to receptors and small molecules, and rotatable bonds were designated for small molecules. To prevent the loss of the interaction force in the conformational search, the hydrogen bond interaction in the cocrystal structure was set as non-rotatable. The grid point spacing was set to be 0.375 Å with a box size of 60 × 70 × 60. For the conformational search, each molecule was given 256 docking conformations using the Lamarckian genetic algorithm (LGA) and the conformation with the highest score was selected as the final conformation. Subsequently, the obtained structural conformation of CCF was fixed in the active cavity of glucosidase. Next, docking of the GA molecule into the active cavity was performed. The binding pattern between guest and enzyme molecules was obtained by selecting the binding conformation with the highest score. Finally, the scoring equation in AutoDocking Tools 1.5.6 was used to calculate the combined free energy of each system. We performed docking analyses according to the above protocols validated by re-docking [36]. We used the same docking method and parameters to dock acarbose to the 3D structure of 3TOP; then, the docked conformation was compared with the crystal structure conformation of acarbose in 3TOP.

### 3.5. Statistical Analysis

Statistical analysis was performed by t-test using SPSS 21.0 software program (Statistical Product and Service Solutions Company, Chicago, IL, USA). A 0.05 level of probability was taken as the minimal level of significance.

## 4. Conclusions

The solubility, dissolution, oral bioavailability and α-glucosidase inhibitory activity of GA were improved through forming cocrystals with glutaric acid and succinimide. The most interesting finding was the binding mechanism between α-glucosidase and the GA-based cocrystal. To the best of our knowledge, this study is the first to demonstrate the changes induced by cocrystals on the binding interaction of API with the targeting protein (here α-glucosidase) using experimental and molecule docking methods. Molecule docking can provide an intuitive method to observe the interaction between inhibitors and enzymes, and give a reasonable explanation of the inhibition mechanism. Many studies have only performed the docking simulation on cocrystal supramolecules; however, no evidence has been found from experimental tests on the corresponding inhibitory activity. Docking conformation between the API-CCF supramolecule and the target protein only is not enough to give the true mechanism of inhibitory activity of the cocrystal. In the present study, based on binding conformations constructed not only on the cocrystal but on free API and CCF and the experimental enzyme inhibitory activity, we deduced that GA-glutaric and GA-succinimide cocrystals were bound to α-glucosidase by different mechanisms. Different supramolecular structures constructed from various CCF molecules may lead to different binding conformations and affinity to α-glucosidase. Obviously, GA was not most valuable α-glucosidase inhibitor with a mM experimental IC_50_ reported above; however, it was a good model that can be used to address the interesting issues of the pharmaceutical cocrystal because it had many cocrystals with different CCFs. Inspired by the synergy mechanism of API and CCF in vitro reported in the present study, screening other GA-based cocrystals and the investigations on synergy action in vivo will be conducted in our next project.

## Figures and Tables

**Table 1:** Activities of GA, glutaric acid and cocrystals.

Sample	IC_50_ (mmoL·L^−1^)
GA	2.79 ± 0.09
Glutaric acid	1.51 ± 0.04
GA-glutaric acid ^**^^△△^	1.04 ± 0.04
GA-succinimide cocrystal ^**^	1.84 ± 0.30

Note: ^**^ *p* < 0.01compared with GA; ^△△^ *p* < 0.01compared with glutaric acid.

## Supplementary Materials

The following are available online. Figure S1: 1H NMR for GA, CCFs and cocrystals (a) GA; (b) CCF (glutaric acid in A and succinimide in B); (c) cocrystal, Table S1: Mean pharmacokinetic parameters of GA and its cocrystals in rats after oral administration (n = 6, $\overset{-}{\chi }$·±S)
