# Supplementary material for: Characterizations and Assays of α-Glucosidase Inhibition Activity on Gallic Acid Cocrystals: Can the Cocrystals be Defined as a New Chemical Entity During Binding with the α-Glucosidase?
Source: Molecules. 2020 Mar 5;25(5):1163. doi: 10.3390/molecules25051163 (PMC7179128; doi:10.3390/molecules25051163)
Supplement: Supplementary file 1 [file molecules-25-01163-s001.pdf]

Supplementary Materials

Table S1 Mean pharmacokinetic parameters of GA and its cocrystals in rats after oral administration (n = 6, $\bar{\chi}$ ±S)

Drugs	C_{max} ($mg \cdot L^{-1}$)	AUC_{0-t} ($mg \cdot h \cdot L^{-1}$)	$AUC_{0-\infty}$ ($mg \cdot h \cdot L^{-1}$)
GA	1.5±0.5	4.1 ±1.2	4.2 ±1.2
GA-glutaric acid cocrystal	2.7±0.6*	7.7±1.0*	7.7±1.0*
GA-succinimide cocrystal	2.2±0.7 ^{ns}	10.6±3.6*	10.8±3.7*

Note: * $p<0.05$, ns= no significance compared with GA.

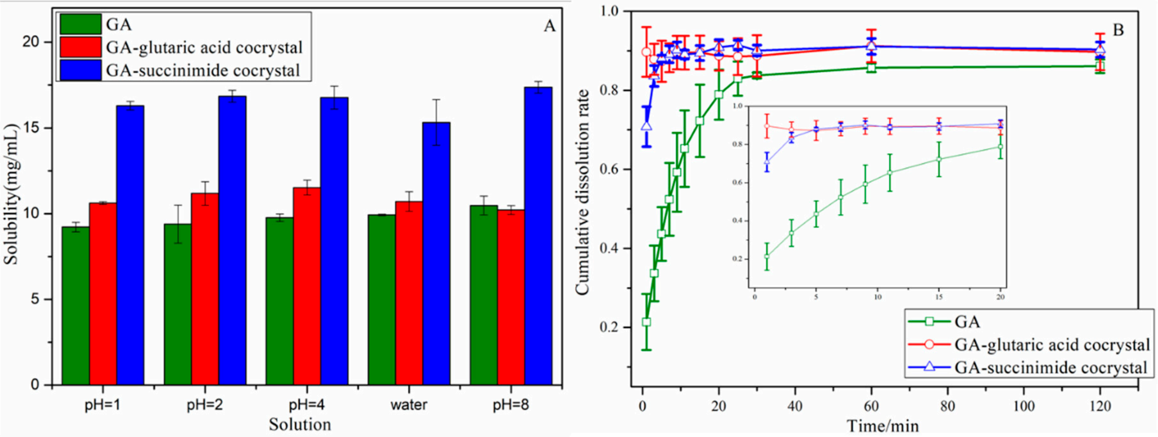

Fig.S1 The solubility (A) and dissolution rate (B) of GA and its cocrystals

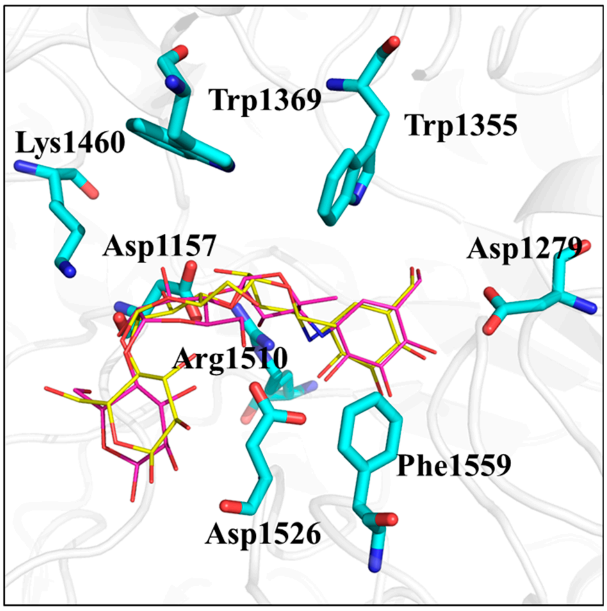

Fig. S2. The re-docking conformations. The docked conformation (pink line) was compared with the crystal structure conformation (yellow line) of acarbose in 3TOP.

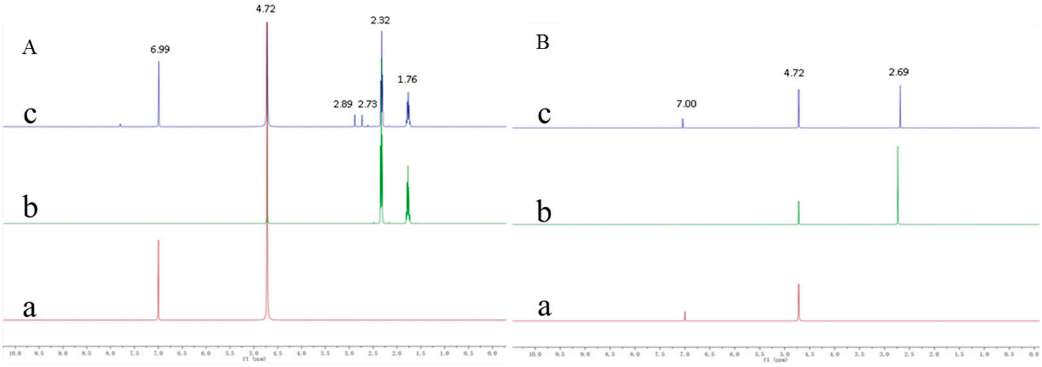

Figure S3 ^{1}H NMR for GA, CCFs and cocrystals (a) GA; (b) CCF (glutaric acid in A and succinimide in B); (c) cocrystal
